# RefNetBuilder: a platform for construction of integrated reference gene regulatory networks from expressed sequence tags

**DOI:** 10.1186/1471-2105-12-S10-S20

**Published:** 2011-10-18

**Authors:** Ying Li, Ping Gong, Edward J Perkins, Chaoyang Zhang, Nan Wang

**Affiliations:** 1 School of Computing, University of Southern Mississippi, Hattiesburg, MS 39406, USA; 2 Environmental Services, SpecPro Inc., San Antonio, TX 78216, USA; 3 Environmental Laboratory, U.S. Army Engineer Research and Development Center, Vicksburg, MS 39180, USA

## Abstract

**Background:**

Gene Regulatory Networks (GRNs) provide integrated views of gene interactions that control biological processes. Many public databases contain biological interactions extracted from experimentally validated literature reports, but most furnish only information for a few genetic model organisms. In order to provide a bioinformatic tool for researchers who work with non-model organisms, we developed RefNetBuilder, a new platform that allows construction of putative reference pathways or GRNs from expressed sequence tags (ESTs).

**Results:**

RefNetBuilder was designed to have the flexibility to extract and archive pathway or GRN information from public databases such as the Kyoto Encyclopedia of Genes and Genomes (KEGG). It features sequence alignment tools such as BLAST to allow mapping ESTs to pathways and GRNs in model organisms. A scoring algorithm was incorporated to rank and select the best match for each query EST. We validated RefNetBuilder using DNA sequences of *Caenorhabditis elegans*, a model organism having manually curated KEGG pathways. Using the earthworm *Eisenia fetida* as an example, we demonstrated the functionalities and features of RefNetBuilder.

**Conclusions:**

The RefNetBuilder provides a standalone application for building reference GRNs for non-model organisms on a number of operating system platforms with standard desktop computer hardware. As a new bioinformatic tool aimed for constructing putative GRNs for non-model organisms that have only ESTs available, RefNetBuilder is especially useful to explore pathway- or network-related information in these organisms.

## Background

Gene regulatory networks (GRNs) offer integrated views of gene interactions that control biological processes. Meanwhile, a number of reverse engineering approaches have been developed to infer GRNs. For instance, Boolean network [1], probabilistic Boolean network [2], modelling algorithms using mutual information (e.g., CLR [3] and ARACNE [4]), and dynamic Bayesian network [5]. The accuracy of computationally inferred GRNs is often evaluated using manually curated pathway or interaction information of model organisms. Such information as functional annotation and relevant biological interactions associated with a particular gene is available from many online resources [6-9]. These public databases contain genetic interactions retrieved from literature with experimental validations, but unfortunately, only a few well-studied model organisms have been curated. The same types of genetic interaction information do not exist for non-model species despite a wealth of transcriptome-wide expressed sequence tags (ESTs) for the specific organisms of interests.

Although experimentally validated interactions among genes or proteins are deposited in the public databases, limitations in accessibility and scalability make retrieving and integrating relevant information difficult. Several bioinformatic toolkits have been developed to extract biological interactions from public databases for well-studied model organisms. For example, BioNetBuilder [10,11] and NetMatch [12] are Cytoscape [13] plug-ins for retrieving, integrating, visualization and analysis of known biological networks. However, these programs cannot be applied to species that have no or limited genetic interaction information. Other tools such as BlastPath [14] and OmicViZ [15], also Cytoscape plug-ins, allow network mapping across species based on sequence homology. But they only map a query species to its closely related model organisms; and have limitations in the number of query genes / proteins. For many less-studied non-model organisms, their related species are often unavailable on the well-annotated model organisms list. Recently, an automatic genome annotation and pathway reconstruction tool named KAAS (KEGG Automatic Annotation Server) was developed for organisms with complete genome sequences [16,17]. To the best of our knowledge, no tools are currently available that provide an integrated environment for building GRNs for less-studied non-model organisms from incomplete genomic or EST sequencing data. This motivates us to develop Reference Network Builder or RefNetBuilder, a cyber-based platform that constructs homologous reference GRNs, to fill this gap.

## Usage

The intended applications of RefNetBuilder include: (1) build putative, reference GRNs/pathways for non-model organisms; (2) provide biological prior knowledge of GRNs that may assist in assessing and improving computational GRN inference models; (3) help to interpret and compare the GRNs reconstructed from wet-lab experiments; and (4) serve as a gene set selection tool for GRN reconstruction because many computational models can only accommodate a limited number of genes (nodes) from high dimensional microarray datasets.

## Methods

**Figure 1:**
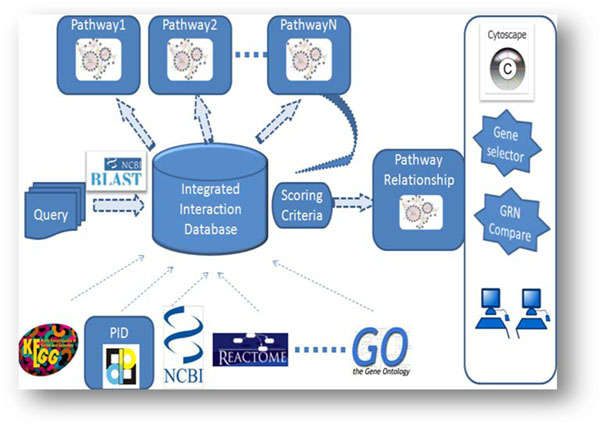
Overview of the RefNetBuilder platform

**Figure 2:**
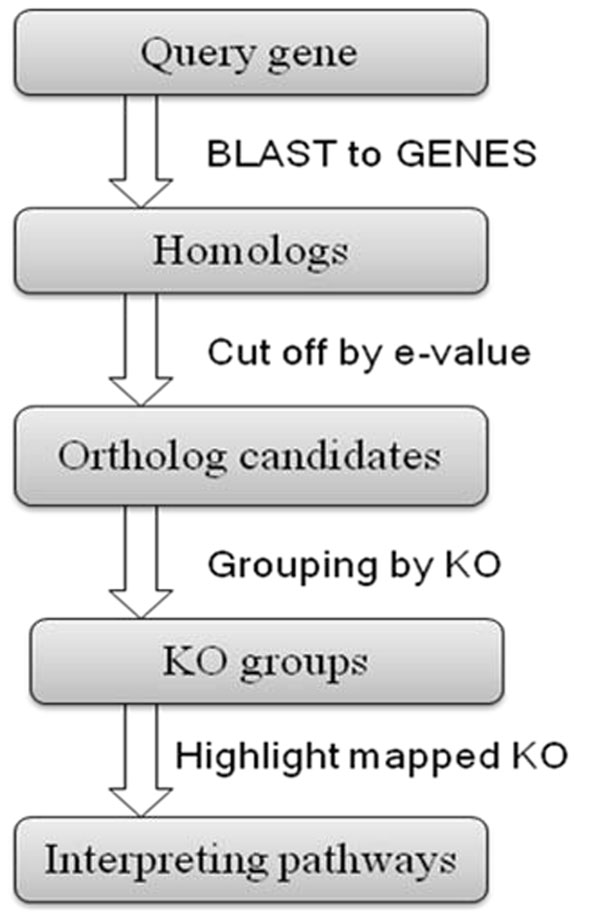
**The work flow in RefNetBuilder.** The KEGG GENES databases are used here for pathway annotation of ESTs.

The platform overview and the work flow of RefNetBuilder are presented in Figures 1 and 2, respectively. Details about the tool development are described as follows:

### Mapping of homologous genes

Homology among proteins and DNA is often concluded on the basis of sequence similarity. The Basic Local Alignment Search Tool (BLAST) [18,19] is one of the most popular and widely-used algorithm for comparing primary biological sequence information, such as the amino-acid sequences of proteins or the nucleotide sequences of DNA. A BLAST search enables comparison of a query sequence with a library or database of sequences. The library sequences that resemble the query sequence above a certain threshold are identified. In RefNetBuilder, the program *blastx* is used, after formatting the database of sequences, to map gene fragments of the query organism to select multiple model organisms in the KEGG (Kyoto Encyclopedia of Genes and Genomes) database. The rationale behind this selection is that many gene structures and functions, as well as pathways, are conserved in evolution. The default settings for the program were used and we limited the maximum target sequences to be one so that the best hit for a query sequence was picked. The cutoff for expect value (*E*-value) was set at 10 by default and the matching sequence that had a higher *E*-value (>10) were considered no statistical similarity. The *E*-value, along with the percentage of identity (*pident*) and the length of the identity (*nident*), was recorded.

### Public databases of genetic interactions

Although many public databases contain information of genetic interactions associated with a particular pathway, pathway annotation is generally sparse for organisms other than human, mouse and rat. Many other organisms with fully sequenced genomes have very limited pathway annotation, which are usually located in dedicated databases that are difficult to retrieve. KEGG [16] is a collection of online databases dealing with genomes, enzymatic pathways, and biochemistry. The KEGG PATHWAY database archives information on molecular interaction networks, such as pathways and complexes, information about genes and proteins generated by genome projects, and information about biochemical compounds and reactions. In RefNetBuilder, all the systematic reference pathways/networks in the KEGG databases have been extracted and loaded into our own pathway annotation database. There are two major categories of reference pathways, namely metabolic pathways and non-metabolic pathways. The non-metabolic pathways capture the perturbed reaction/interaction networks for genetic information processing, environmental information processing, other cellular processes, and human diseases. The molecular network shown in each pathway map is a graph consisting of nodes (e.g., genes, proteins, small molecules, etc.) and edges (reactions, interactions and relations). In general, if two genes in the pathway map are connected with an edge, they are considered to have a regulatory relationship. Each gene extracted from the KEGG GENES database is assigned a unique KEGG Orthology (KO) identifier (KOID). The KO entry represents an ortholog group that is linked to a gene product in the KEGG pathway diagram. Thus, the BLAST scores between a query sequence and the reference sequence set from the KEGG GENES database are computed, and homologs are found in the reference set (Figure 2).

### RefNetBuilder: Reference networks for non-model organisms

After BLAST between query genes and the reference gene set from KEGG GENES database, homologs are found for each query sequence. Then, homologs ranked above the threshold are selected as ortholog candidates based on the BLAST score. Ortholog candidates are divided into KO groups according to the annotation of the KEGG GENES database and each query sequence is mapped with the corresponding KO group (Figure 2).

### Interpretation and integration of networks

Based on the results of mapping between query sequences and KO reference genes from the KEGG GENES database, all the reference pathways extracted from the KEGG database are interpreted by highlighting those KO reference genes if they are mapped to a query sequence from the non-model organism. That is, for each pathway map, the node (representation of ortholog gene) is highlighted in the red colour if it is the best hit for a query sequence, and gene names are replaced by its corresponding KO group identification. The rest of the structure on the map remains the same as in the original map from KEGG database. By using the KGML-ED tool [20], the customized interpretation of pathway maps that include mapping information of query gene and KO reference gene are generated and can be used as a graphical representation of reference GRNs/pathways for the query non-model organism.

## Results and discussion

We first tested the accuracy of RefNetBuilder by reassigning KO identifiers to the *Caenorhabditis elegans* (nematode) genes queried against seven other model species curated in the KEGG GENES database. The seven organisms are *Anopheles gambiae* (mosquito), *Apis mellifera* (honey bee), *Drosophila melanogaster* (fruit fly), *Homo sapiens* (human), *Mus musculus* (mouse), *Rattus norvegicus* (rat) and *Schistosoma mansoni* (flatworm). Currently, 3913 *C. elegans* genes are annotated with a KO identifier number (KOID). The test results (see **Additional file**1) show that RefNetBuilder was able to assign each of the *C. elegans* gene a KOID with a 70.9% accuracy, i.e., 2773 assigned KOIDs matching the original KEGG curated KOIDs. This accuracy rate is comparable to the 62.5% ~90.1% sensitivity reported for KAAS by querying against a representative set of model species [17].

To demonstrate the functionality and features of RefNetBuilder, we used a non-model organism, the earthworm *Eisenia fetida*, as an example. A total of 43,803 *E. fetida* ESTs were queried against the above-mentioned eight model organisms. After processing through RefNetBuilder, 9,187 of these ESTs were assigned to 3,134 unique KOIDs that were mapped to 267 pathways out of the entire 317 KEGG pathways (see **Additional file**2). A subset of 2,574 earthworm ESTs identified as differentially expressed genes in response to chemical perturbations (unpublished data) was also annotated using RefNetBuilder. Results (see **Additional file**3) show that 604 of these ESTs were assigned to 450 unique KOIDs that belong to 226 KEGG pathways (88 metabolism and 138 non-metabolism pathways), with 218 ESTs being mapped to metabolic pathways, 460 to non-metabolic pathways, and 74 to both.

**Figure 3:**
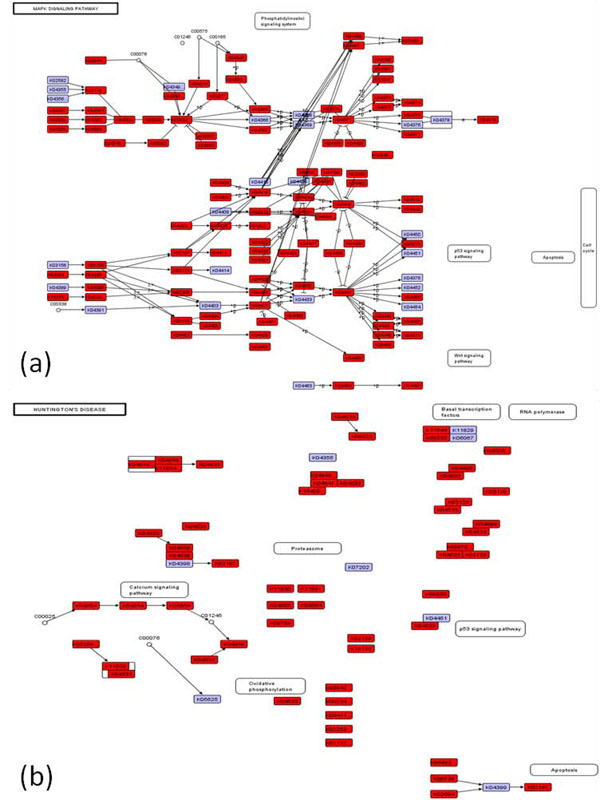
**Graphical reference pathways generated by RefNetBuilder.** Two example pathways for the earthworm *Eisenia fetida*: (a) MAPK signaling; and (b) Huntington’s disease.

Figure 3 shows two KEGG pathways mapped with *E. fetida* ESTs (also see **Additional file**2). A total of 327 mapped earthworm ESTs are present in the MAPK Signalling Pathway, corresponding to 181 unique KO orthologs in the KEGG database (Figure 3a). Similarly, 372 earthworm ESTs are mapped to the Huntington’s Disease Pathway matching 147 unique KO orthologs (Figure 3b). Therefore, two earthworm ESTs match approximately one KO gene.

The above derived pathway information is currently being used for computational inference of GRNs from a large earthworm microarray dataset. Meanwhile, other curated pathway databases such as the Pathway Interaction Database (PID) [21], Reactome [22] and the BioCyc Tier 1 databases [23] are being added to the RefNetBuilder platform (Figure 1). This platform has the flexibility to expand and include more interaction information as it becomes available in the future.

## Conclusions

Here we presented the development of RefNetBuilder, a new tool aimed for constructing GRNs for non-model organisms that have only ESTs available. Researchers who wish to explore pathway- or network-related bioinformatic information in these organisms may find this tool especially useful.

## Availability and requirements

Project name: The RefNetBuilder Platform

Project Available at: http://orca.st.usm.edu/cbbl/refnet

Operating system(s): Windows XP, Vista(x86), Vista(x64), Linux, MacOS Programming languages: Perl

Other requirements: MySQL Server, ActivePerl, Blast

Any restrictions to use by non-academics: None

## Competing interests

The authors declare that they have no competing interests.

## Authors' contributions

NW and PG conceived the project. EJP, PG and CZ coordinated the study. YL and NW developed and implemented the platform. YL, NW and PG performed in-depth analysis of results. YL drafted the manuscript. PG, EJP, NW and CZ revised the manuscript. All authors read and approved the final manuscript.

## Supplementary Material

Additional file 1 **Supplementary Table 1** Test results of *C. elegans* genes queried against seven other model species, showing the RefNetBuilder assigned KOIDs in comparison with KEGG curated KOIDs for *C. elegans*. Click here for file

Additional file 2 **Supplementary Table 2** Example: Mapping 43,803 *E. fetida* (earthworm) ESTs to KEGG pathways using RefNetBuilder. Click here for file

Additional file 3 **Supplementary Table 3** Mapping results of 2,574 *E. fetida* ESTs, a subset of differentially expressed transcripts derived from an unpublished earthworm microarray study. Click here for file
